# MALDI-TOF MS Biomarker Detection Models to Distinguish RTX Toxin Phenotypes of *Moraxella bovoculi* Strains Are Enhanced Using Calcium Chloride Supplemented Agar

**DOI:** 10.3389/fcimb.2021.632647

**Published:** 2021-03-16

**Authors:** Matthew M. Hille, Michael L. Clawson, Aaron M. Dickey, Justin H. Lowery, John Dustin Loy

**Affiliations:** ^1^School of Veterinary Medicine and Biomedical Sciences, Institute for Agriculture and Natural Resources, University of Nebraska-Lincoln, Lincoln, NE, United States; ^2^U.S. Meat Animal Research Center, United States Department of Agriculture, Agricultural Research Service, Clay Center, NE, United States

**Keywords:** MALDI-TOF MS, *Moraxella bovoculi*, infectious bovine keratoconjunctivitis, biomarker model, RTX toxin, Moraxella bovis

## Abstract

*Moraxella bovoculi* is the bacterium most often cultured from ocular lesions of cattle with infectious bovine keratoconjunctivitis, also known as bovine pinkeye. Some strains of *M. bovoculi* contain operons encoding for a repeats-in-toxin (RTX) toxin, which is a known virulence factor of multiple veterinary pathogens. We explored the utility of MALDI-TOF MS and biomarker detection models to classify the presence or absence of an RTX phenotype in *M. bovoculi*. Ninety strains that had undergone whole genome sequencing were classified by the presence or absence of complete RTX operons and confirmed with a visual assessment of hemolysis on blood agar. Strains were grown on Tryptic Soy Agar (TSA) with 5% sheep blood, TSA with 5% bovine blood that was supplemented with 10% fetal bovine serum, 10 mmol/LCaCl_2_, or both. The formulations were designed to determine the influence of growth media on toxin production or activity, as calcium ions are required for toxin secretion and activity. Mass spectra were obtained for strains grown on each agar formulation and biomarker models were developed using ClinProTools 3.0 software. The most accurate model was developed using spectra from strains grown on TSA with 5% bovine blood and supplemented with CaCl_2_, which had a sensitivity and specificity of 93.3% and 73.3%, respectively, regarding RTX phenotype classification. The same biomarker model algorithm developed from strains grown on TSA with 5% sheep blood had a substantially lower sensitivity and specificity of 68.0% and 52.0%, respectively. Our results indicate that MALDI-TOF MS biomarker models can accurately classify strains of *M. bovoculi* regarding the presence or absence of RTX toxin operons and that agar media modifications improve the accuracy of these models.

## Introduction

Infectious bovine keratoconjunctivitis (IBK) is the most common ocular disease in cattle (Brown et al., 1998). IBK has a substantial economic impact including costs associated with treatment as well as decreased weight gain in affected animals and impacts animal welfare by causing pain and blindness (Killinger et al., 1977; Dewell et al., 2014). *Moraxella bovis* (*M. bovis*) is the only bacterium that has reproduced IBK-like lesions in a variety of experimental models (Henson and Grumbles, 1960; Aikman et al., 1985). Other bacteria are often associated with IBK, but so far none have produced disease experimentally. The most notable and frequently isolated of these associated bacteria is *Moraxella bovoculi* (Angelos et al., 2007b). While *M. bovoculi* has been unsuccessful at inducing IBK experimentally (Gould et al., 2013), it is more frequently isolated from IBK lesions when compared to *M. bovis*, using both aerobic culture and molecular detection techniques (Loy and Brodersen, 2014; Zheng et al., 2019). Despite the lack of proven causation for *M*. bovoculi in IBK to date, in 2017 the USDA approved the first conditionally licensed *M. bovoculi* based vaccine product to be marketed for the prevention of IBK (USDA CVM code: 2A77.00, Addison Biological Laboratory). Recently, whole genome sequencing of *M. bovoculi* has revealed a large degree of diversity within the species that led to the characterization of two distinct genotypes (genotype 1 and genotype 2) separated by over 23,000 single nucleotide polymorphisms (Dickey et al., 2018). To date, only genotype 1 *M. bovoculi* have been isolated from IBK lesions while both genotypes have been recovered from animals without clinical signs.

Strains of *M. bovis* have been shown to produce an exotoxin belonging to the repeats-in-toxin (RTX) class of exotoxins that is cytopathic to bovine erythrocytes and neutrophils (Clinkenbeard and Thiessen, 1991; Angelos et al., 2001). In *M. bovis*, this RTX toxin, encoded by *mbxA* within an operon, is often referred to by several names including cytolysin, hemolysin, or cytotoxin. RTX toxins are known virulence factors in a variety of veterinary pathogens, including species within the family *Pasteurellacea* (Linhartova et al., 2018). A well-studied example of this is the leukotoxin produced by *Mannheimia haemolytica* (Frey, 2019). These RTX toxins are secreted in a calcium-dependent manner *via* a type I secretion system (T1SS) responsible for translocating the toxin from the cytosol to the exterior (Linhartova et al., 2018) Like *M. bovis*, some *M. bovoculi* strains also contain a complete RTX operon that produces an RTX toxin, cytotoxin A encoded by *mbvA* (Angelos et al., 2007a). Like *mbxA* produced by *M. bovis, mbvA* of *M. bovoculi* is responsible for hemolytic and lytic activity on bovine cells (Cerny et al., 2006; Angelos et al., 2007b). Within *M. bovoculi*, only the disease associated genotype 1 strains have been shown to possess the RTX operon, although not all do (Dickey et al., 2018). Besides RTX toxins, both *M. bovis* and *M. bovoculi* express a type IV pilus, a known virulence factor in other bacterial pathogens (Angelos et al., 2021). These similar potential virulence factors between the two *Moraxella* species highlight the relevance of continued investigation into the diversity within *M. bovoculi* and how this diversity may impact IBK pathogenesis.

Matrix-assisted laser desorption/ionization time-of-flight mass spectrometry (MALDI-TOF MS) is an approach that is increasingly being applied to the identification of prokaryotes and eukaryotes. (Seng et al., 2009; Clark et al., 2013; Khot and Fisher, 2013; Karger et al., 2019). MALDI-TOF MS has replaced or supplemented more traditional biochemical identification methods as it is faster and increasingly more accurate as databases become mature. Using spectrum profiles generated from MALDI-TOF MS, biomarker models have been developed that are capable of differentiating subspecies and genotypes within a given bacterial species (Loy and Clawson, 2017; Mani et al., 2017; Perez-Sancho et al., 2018). Recently, a MALDI-TOF biomarker model was developed that accurately distinguishes *M. bovoculi* genotypes 1 and 2 (Hille et al., 2020). This model allows for the screening of strains for potential disease associated genotypes without the need for genome sequencing.

Genotype 1 *M. bovoculi* strains isolated from IBK associated eyes have been found with a higher frequency of the RTX operon than those isolated from unaffected eyes (Dickey et al., 2018). While it can be suspected based on a hemolytic phenotype, confirming the presence or absence of an RTX operon requires PCR or genomic sequencing (Angelos et al., 2003). In this study we evaluated the utility of MALDI-TOF MS biomarker models to accurately classify *M. bovoculi* strains regarding the presence or absence of an RTX operon among strains that had previously undergone whole genome sequencing and thus whose RTX operon status was known. Such a model would allow for an additional screening tool to characterize strains more likely to represent disease associated strains.

In addition to traditional tryptic soy agar (TSA) with 5% sheep blood agar culture conditions, we also compared biomarker model accuracies using three additional growth agar formulations to determine if agar formulation could improve model accuracy. The hemolytic activity of hemolysin produced by *M. bovis* has been shown to decrease when extracellular calcium is rendered unavailable (Clinkenbeard and Thiessen, 1991; Billson et al., 2000). Calcium has also been shown to promote efficient post translational modification and excretion of RTX toxins from the cell *via* the T1SS in *Bordetella pertussis* (Bumba et al., 2016). We hypothesized calcium may be a limiting factor in the production of RTX for *M. bovoculi* using the traditional culture conditions. Additionally, in the authors’ experience, *Moraxella* sp. isolated from cattle grow subjectively better on agar that utilizes bovine red blood cells vs traditional sheep’s blood agar.

## Methods

### Bacterial Strains

The 90 *M. bovoculi* strains used in this study had previously been identified to the species level using both PCR and MALDI-TOF MS techniques as previously described (Loy and Brodersen, 2014; Robbins et al., 2018). The strains also underwent whole genome sequencing which enabled characterization to the genotype level as well as the presence or absence of an RTX operon element (Dickey et al., 2018). Included were 45 genotype 1, RTX negative and 45 genotype 1, RTX positive strains obtained from a total of 18 different states within the US (**Table 1**). Hemolytic activity on blood agar containing 5% sheep blood was confirmed for all RTX positive strains and was absent in all RTX negative strains. All 90 strains were used in the models developed from TSA with 5% sheep erythrocytes and a subset of 70 strains were used in the models developed from TSA with 5% bovine erythrocytes.

**Table 1:** The biomarker model group, RTX status, and state of origin for the 90 *M. bovoculi* strains used in this study.

Group	Strain Number	RTX +/-	State
Model Generation	57909	–	Nebraska
	58026	–	Oklahoma
	58036	–	Iowa
	58058	–	Nebraska
	58065	–	Indiana
	58079	–	Kansas
	58094	–	Nebraska
	58122	–	Nebraska
	60479	–	Nebraska
	68507	–	Nebraska
	57851	+	Montana
	57855	+	Ohio
	57904	+	California
	57917	+	Nebraska
	57922	+	Indiana
	58001	+	Montana
	58015	+	Illinois
	58027	+	Virginia
	58080	+	Minnesota
	58119	+	Nebraska
Model Validation	57861	–	South Dakota
	57876	–	Nebraska
	57923	–	Minnesota
	58034	–	Nebraska
	58054	–	Kansas
	58075	–	Nebraska
	58090	–	Illinois
	60476	–	Nebraska
	68485	–	Nebraska
	68511	–	Nebraska
	57860	+	Iowa
	57870	+	Nebraska
	57884	+	Indiana
	57891	+	Wisconsin
	57993	+	Nebraska
	58030	+	Kansas
	58035	+	Illinois
	58053	+	Montana
	58063	+	Minnesota
	58088	+	Minnesota
Model Classify	57881	–	Nebraska
	57883	–	Nebraska
	58028	–	South Dakota
	58047	–	Virginia
	58067	–	Nebraska
	58086	–	Virginia
	58123	–	Iowa
	60481	–	Nebraska
	68486	–	Nebraska
	68512	–	Nebraska
	68513	–	Nebraska
	68542	–	Nebraska
	68552	–	Nebraska
	68554	–	Nebraska
	68555	–	Nebraska
	58029*	–	Wisconsin
	58037*	–	Nebraska
	58044*	–	Oklahoma
	58055*	–	Kansas
	58091*	–	Nebraska
	58108*	–	Nebraska
	60478*	–	Nebraska
	68528*	–	Nebraska
	68529*	–	Nebraska
	68541*	–	Nebraska
	57854	+	Nebraska
	57863	+	Indiana
	57865	+	Nebraska
	57879	+	Oklahoma
	57894	+	Kansas
	57903	+	California
	57905	+	Iowa
	57906	+	Montana
	57918	+	North Dakota
	57919	+	Washington
	58009	+	Montana
	58016	+	Illinois
	58039	+	Kansas
	58097	+	South Carolina
	58101	+	Ohio
	57857*	+	Texas
	57871*	+	Nebraska
	57873*	+	Nebraska
	57878*	+	Nebraska
	57887*	+	Wisconsin
	57892*	+	Indiana
	57894*	+	Kansas
	58010*	+	Tennessee
	58011*	+	Illinois
	58069*	+	Nebraska

All strains were used in the development of models using TSA + 5% sheep erythrocytes. Strain numbers with an asterisk signify strains omitted from the TSA + 5% bovine erythrocyte model development portion of this study.

### Culture Conditions

From a library of frozen stocks, *M. bovoculi* strains were plated on either TSA(Becton, Dickinson & Company, Sparks, MD) with 5% sheep blood (Remel, Lenexa, KS) or TSA with 5% defibrinated bovine blood (Colorado Serum Company, Denver, CO). Additionally, the agars using bovine blood were supplemented with either 10% fetal bovine serum (Colorado Serum Company), 10 mmol/L CaCl_2_ (Fisher Scientific, Waltham, MA), or both. After the strains were plated they were incubated in 5% CO_2_ at 37° C for 24 hours prior to being passed once on the same agar formulation for an additional 24 hours prior to MALDI-TOF MS analysis.

### MALDI-TOF MS

MALDI-TOF MS spectra were obtained for each of the strains listed in **Table 1** according to the manufacturer’s recommendation using the formic acid-ethanol extraction method that has previously been described (Khot et al., 2012; Hille et al., 2020). Spectra were collected on a linear MALDI-TOF MS (Bruker microflex, Bruker Daltonik, Billerica, MA) using settings and calibrations as described previously (Hille et al., 2020). For the sheep’s blood media models, strains were spotted three times on the target plate and analyzed once for each spot well resulting in three spectra per strain. For the bovine blood models, the strains were spotted five times each and analyzed twice per well resulting in 10 spectra per strain. This yielded a total of 970 unique spectra being analyzed for this study.

### Biomarker Models

ClinProTools 3.0 software (Bruker Daltonik) was used to analyze the spectra for the presence of RTX phenotype specific, discriminatory peaks. To develop biomarker models, three spectra classification algorithms were used that included support vector machine (SVM), genetic algorithm (GA), and quick classifier (QC) methods. *M. bovoculi* strains were randomly assigned to separate groups according to RTX status to develop the biomarker models which included model generation, model validation, and model classify groups (**Table 1**). Spectra from the model generation groups were input into ClinProTools 3.0 software (Bruker Daltonik) according to their known RTX status to develop the models. The model validation step used spectra that were input with the RTX status known to the software as a test of accuracy of the developed models. Finally, the classification step involved inputting spectra with RTX status unknown to the software and allowing the models to classify them. The accuracy of the models to classify spectra was then calculated manually based on individual spectra as well as using the majority classification from a strain’s spectra profile. When the study was expanded to assess different agar formulations, the number of isolates used in the model classification groups was reduced by 10 for each genotype to minimize the extra culture time and computational power required to do the study yet still allow for accuracy comparisons across all models. The isolates removed were chosen at random and resulted in a total of 70 isolates being used for the bovine blood agar portion of the study as opposed to the 90 isolates used in the sheep blood agar portion. When a model classified half of the spectra from a given strain as RTX – and half of the spectra as RTX +, this was counted as an incorrect classification by the model. Sensitivity, specificity, negative predictive value, and positive predictive value were also calculated manually using the genomic sequencing RTX classification as the gold standard.

## Results

### TSA + 5% Sheep Blood SVM Model

The SVM method proved the most accurate for this study overall and is the focus of the remainder of the paper. The parameters and accuracy of all models developed in this study across all culture conditions and biomarker model development methods are included as **supplementary material** (**Supplementary Table 1**). The SVM model developed using TSA + 5% sheep blood yielded individual spectra classification accuracies of 50.7% for RTX – strains and 66.6% for RTX + strains (**Table 2**). In terms of the presence or absence of RTX, these statistics would correlate to specificity and sensitivity, respectively, when interpreting the model result as a diagnostic assay. When all spectra for an individual strain were classified, and the majority model classification was used for the final strain phenotype interpretation, these accuracy values increased slightly to 52% for RTX -, and 68% for RTX + strains. This equates to a negative predictive value of 61.9% and a positive predictive value of 58.6% when using the majority classification.

**Table 2:** Accuracy of SVM models developed in this study using TSA + 5% sheep blood and TSA + 5% bovine blood supplemented with CaCl_2_.

Culture Conditions	TSA + 5% sheep rbc	TSA + 5% bovine rbc + 10 mmol/L CaCl_2_
Recognition	100%	100%
Cross Validation	91.69%	99.23%
RTX – External Validation	23.3%	67%
RTX + External Validation	83.3%	83%
RTX – Classify per spectra	50.7%	67.3%
RTX + Classify per spectra	66.6%	90%
RTX - Classify majority spectra	52%	73.3%
RTX + Classify majority spectra	68%	93.3%
NPV: Classify majority spectra	61.9%	91.7%
PPV: Classify majority spectra	58.6%	77.8%

NPV, negative predictive value; PPV, positive predictive value.

### TSA + 5% Bovine Blood + 10 mmol CaCl_2_ SVM Model

**Figure 1:**
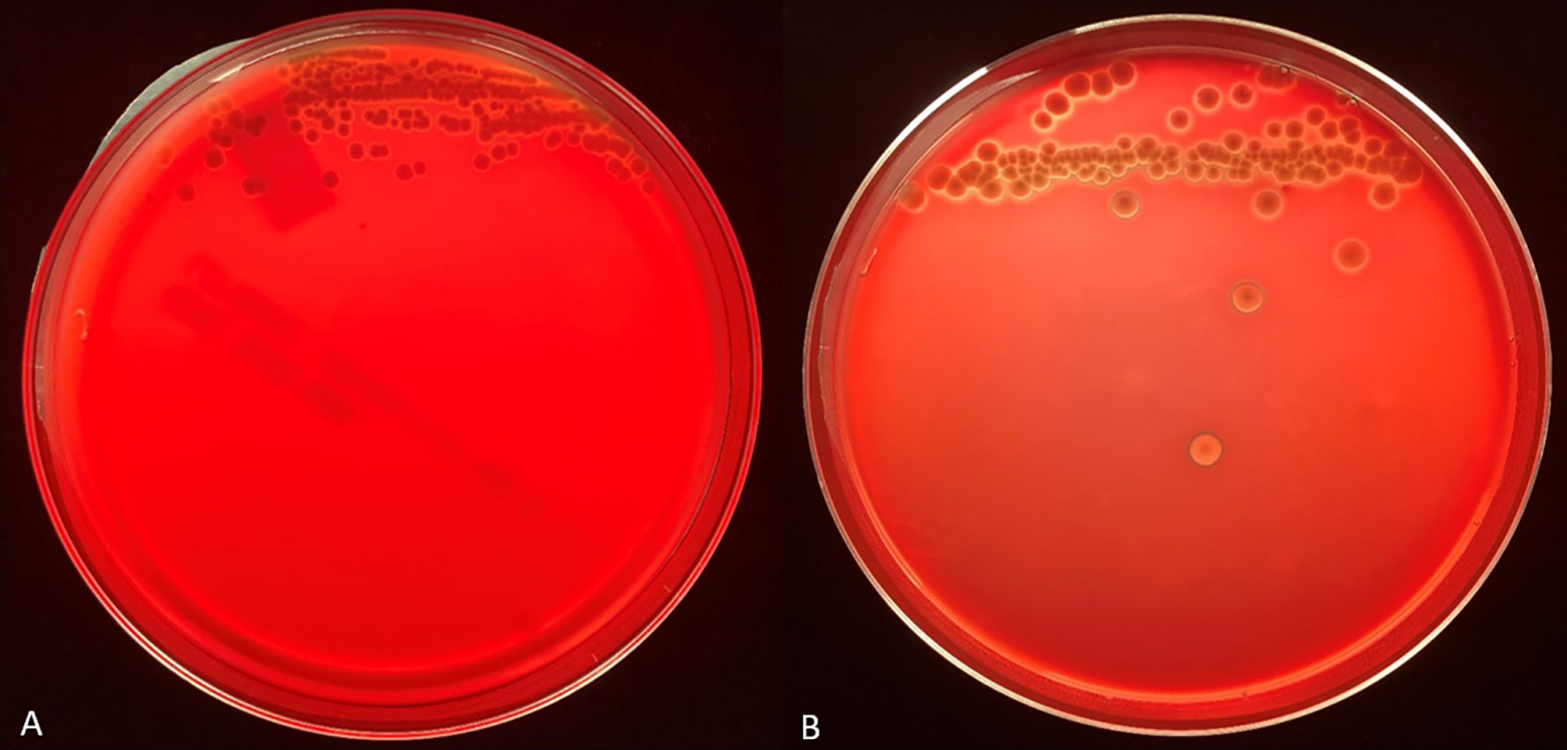
Representative backlit blood agar plates streaked with strain #57905 after 48 hours incubation in 5% CO_2_ at 37°C on TSA with 5% sheep blood **(A)** and TSA with 5% bovine blood and 10 mmol/L CaCl_2_ **(B)**. Care was taken to ensure the thickness of the bovine blood agar within the petri dish was like that of the commercial sheep blood agar.

**Figure 2:**
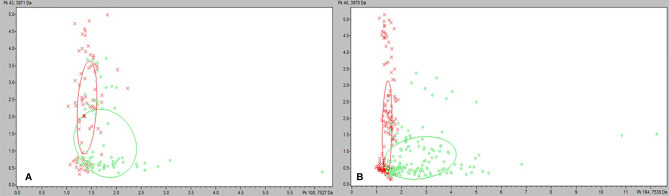
ClinProTools 3.0 2-D plots incorporating the two most common and highest weighted peaks in the study. **(A)** *M. bovoculi* strains grown on TSA + 5% sheep blood. **(B)** *M. bovoculi* strains grown on TSA + 5% bovine blood with 10 mmol/L CaCl_2_. Red X: RTX – strains. Green circle: RTX + strains.

**Figure 3:**
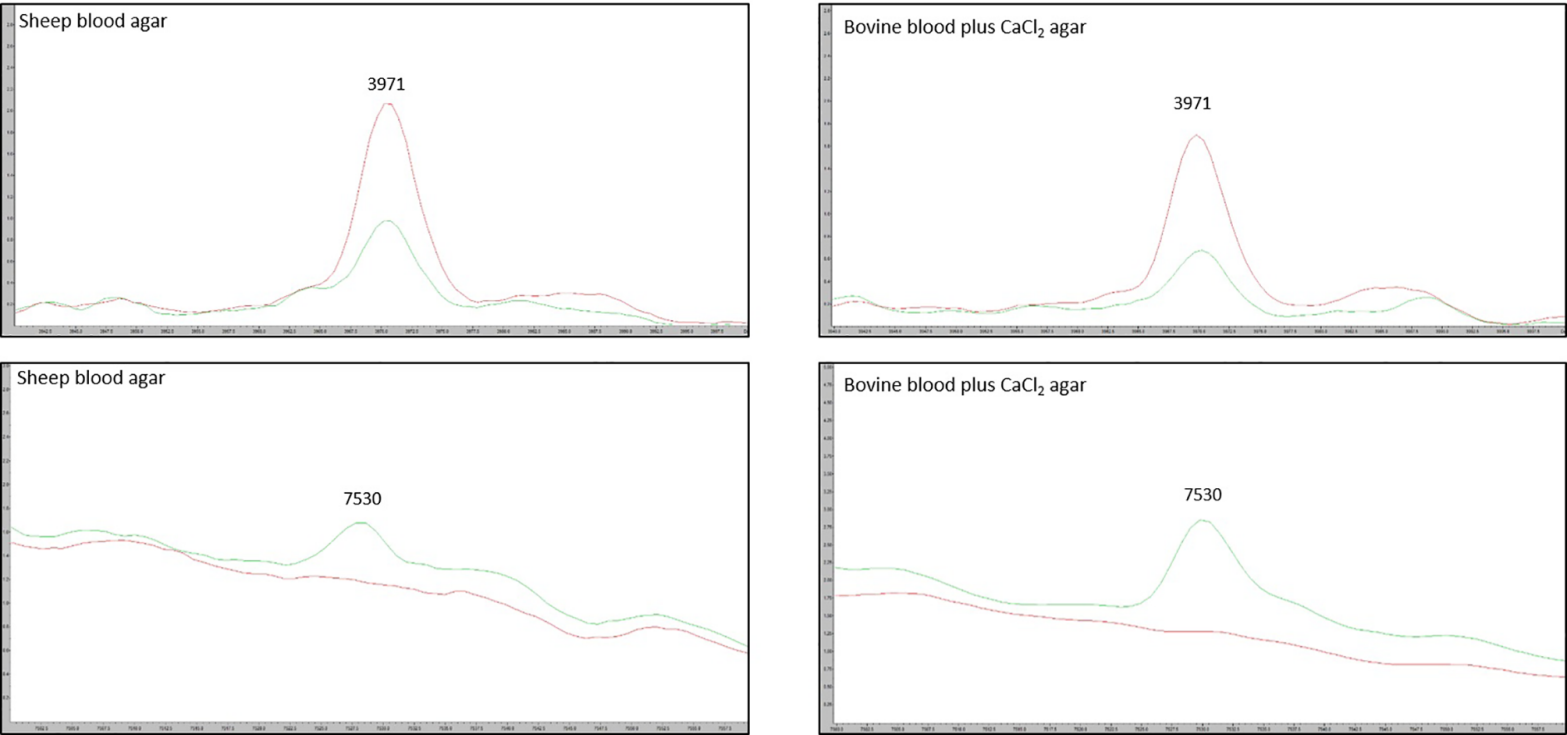
Spectra of the two most discriminatory peaks 3971 m/z and 7530 m/z, compared between the sheep blood agar and bovine blood agar plus CaCl_2_ formulations. Red line: average RTX – spectra for the group of model classify strains. Green line: average RTX + spectra for the group of model classify strains.

We tested the same MALDI-TOF MS biomarker model development methods using strains grown on TSA + 5% bovine blood supplemented with either 10% fetal bovine serum (FBS), 10 mmol/L CaCl_2_, or both. The addition of CaCl_2_ alone to the bovine blood agar resulted in the most accurate model (**Table 2**). The two most common peaks incorporated into the models in this study had m/z values of at or very near 3971 and 7530 (**Supplementary Table 1**). Using these peaks, when we examine 2-dimensional plots within ClinProTools 3.0, the different culture conditions show more defined clustering and less overlap in the spectra of strains grown on bovine blood plates with CaCl_2_ when compared to those grown on the sheep blood plates (**Figure 2**). The models developed incorporating FBS are summarized in the **Supplementary Material** (**Supplementary Table 1**). Using the bovine blood agar and 10 mmol/L CaCl_2_, the specificity and sensitivity increased to 73.3% and 93.3% respectively (**Table 2**). Additionally, the negative predictive value and positive predictive value increased to 91.7% and 77.8% respectively. All the CaCl_2_ supplemented bovine blood plates displayed subjectively larger colonies with a more visually prominent zone of hemolysis suggesting either an overall increase in RTX production per bacterial cell, an increase in RTX hemolytic activity, an increase in cellular division, or a combination of the three (**Figure 1**). The most discriminatory peaks incorporated into the models in this study often had subtle differences between RTX groups that were not always easily discernible by visually examining the spectra alone, regardless of the agar formulation used (**Figure 3**).

## Discussion

Here we have described a group of biomarker models developed using ClinProTools 3.0 software that are capable of correctly phenotyping most *M. bovoculi* genotype 1 strains for RTX. Across all models, the RTX phenotype specificity ranged from 26.7% - 86.7% while the sensitivity ranged from 46.7% - 93.3%. The most accurate model overall was the SVM model developed using strains grown on TSA + 5% bovine blood supplemented with 10 mmol/L CaCl_2_, which had a specificity and sensitivity of 73.3% and 93.3% respectively. Regardless of the biomarker model algorithm used, the usage of agar incorporating bovine blood and CaCl_2_ substantially outperformed sheep blood agar. Since secretion of RTX toxins utilizes a calcium-dependent T1SS, the extra calcium may result in an overall increase in RTX production, although confirming this would require more investigation and was outside the scope of this study. Additionally, mammalian erythrocytes vary in the composition of their membranes (de and Van Deenen, 1961), and the species of erythrocyte has shown to affect activity of other toxins (Bhakdi et al., 1984). This increase in hemolysis may increase nutrient or other factor availability that enhances model performance.

MALDI-TOF MS biomarker models often highlight discriminatory peaks between groups of spectra that are different enough in their m/z value that they can be discerned even without the use of computer models. For instance, within *M. bovoculi* a strong peak at 9057 m/z is specific for genotype 1 whereas a peak at 6550 m/z is specific for genotype 2 (Hille et al., 2020). Having strong discriminatory peaks such as these allows those without ClinProTools 3.0 software to differentiate groups by manually examining spectra. This was not the case for the current study and highlights the need for biomarker models and their algorithmic approach to analysis when spectra peak differences may be subtle between groups.

The SVM model developed in this study using bovine blood agar and CaCl_2_ provides an efficient method of RTX phenotyping for *M. bovoculi* without the need for PCR or genomic sequencing. A negative predictive value of 91.7% means that RTX – strains can be classified accordingly with acceptable accuracy. While the importance of RTX toxins in the pathogenesis of IBK is not fully known, they are regarded as likely important virulence factors and RTX + strains are overrepresented in cases of IBK (Angelos et al., 2007a; Dickey et al., 2018). The ability to classify RTX – strains in this manner may prove beneficial in the formulation of autogenous vaccines for IBK as this will allow vaccine manufacturers to eliminate any RTX – strains from consideration and include only strains that are more likely to represent disease associated, RTX + strains within the vaccine formulation. With hemolysis shown to be RTX mediated in *M. bovis*, an RTX phenotyping MALDI-TOF MS biomarker model for *M. bovis* analogous to the one described here for *M. bovoculi*, would provide the same utility for vaccine formulations that choose to include *M. bovis*. Developing an *M. bovis* model would benefit from a library of sequenced *M. bovis* isolates whose RTX status is defined, unless hemolytic activity alone was used to assume RTX status. Here, we focused solely on *M. bovoculi* given our in-house library of previously sequenced isolates in order to examine the biomarker phenotyping proof-of-concept since the known presence of RTX components allowed us to avoid using hemolysis alone to classify RTX presence. In addition to its relevance to IBK, this study also serves as a blueprint of methods and proof-of-concept for utilizing MALDI-TOF MS spectra and biomarker models to distinguish strains of bacteria based on their ability to produce secreted exotoxins. Such methods could prove beneficial in differentiating other bacterial pathogens of both humans and animals that possess secreted exotoxins as virulence factors. Beyond being a proof-of-concept, the methods described here also reduce the time required to classify a *M. bovoculi* isolate by RTX status based solely on hemolysis. The gross appearance of hemolysis is often not readily apparent until 48 hours of growth while the MALDI-TOF MS biomarkers models we developed here use colonies that were grown for only 24 hours.

A major limitation for this study is the inability to assure only a single variable, in this case RTX presence or absence, differentiates the groups of isolates. It is possible that some of the peaks incorporated into the models developed here represent isolate components unrelated to RTX itself. We have mitigated the likelihood of this limitation affecting the overall study interpretation by: 1) utilizing only genotype 1 strains for the entire study, and 2) incorporating a large number of isolates and spectra within each group, and 3) utilizing geographically diverse populations of isolates for both RTX status groups.

## Data Availability Statement

The original contributions presented in the study are included in the article/**Supplementary Material**. Further inquiries can be directed to the corresponding author.

## Author Contributions

MH performed MALDI-TOF analysis, developed the biomarker models, wrote the original manuscript draft and edited the manuscript. MC and AD performed genomic sequencing analysis on the bacterial strains and edited the manuscript. JHL performed MALDI-TOF analysis. JDL conceptualized the study, supervised the project, and edited the manuscript. All authors contributed to the article and approved the submitted version.

## Funding

This project was funded by the Nebraska Experiment Station with funds from the Animal Health and Disease Research (section 1433) capacity funding program (accession 1017646) through the USDA National Institute of Food and Agriculture. The use of product and company names is necessary to accurately report the methods and results; however, the United States Department of Agriculture (USDA) neither guarantees nor warrants the standard of the products, and the use of names by the USDA implies no approval of the product to the exclusion of others that may also be suitable. The USDA is an equal opportunity provider and employer.

## Conflict of Interest

The authors declare that the research was conducted in the absence of any commercial or financial relationships that could be construed as a potential conflict of interest.
