# Supplementary material for: MALDI-TOF MS Biomarker Detection Models to Distinguish RTX Toxin Phenotypes of Moraxella bovoculi Strains Are Enhanced Using Calcium Chloride Supplemented Agar
Source: Front Cell Infect Microbiol. 2021 Mar 16;11:632647. doi: 10.3389/fcimb.2021.632647 (PMC8007961; doi:10.3389/fcimb.2021.632647)
Supplement: Supplementary file 1 [file Table_1.docx]

|  |  | **SVM** | **GA** | **QC** |
| --- | --- | --- | --- | --- |
|  |  |  |  |  |
|  | Peaks Used (m/z) | 2163^0.98^ | 3970^1.73^ | 3484 |
|  |  | 2243^1.00^ | 2163^0.55^ | 3970 |
|  |  | 3970^1.93^ | 3030^0.66^ | 6405 |
|  |  |  | 6955^0.66^ | 6621 |
|  |  |  | 2243^0.48^ | 6970 |
|  |  |  |  | 7010 |
| **TSA + 5% sheep blood** |  |  |  |  |
|  | Recognition Capability | 100% | 100% | 91.67% |
|  | Cross Validation | 91.69% | 98.36% | 88.30% |
|  | RTX – External Validation | 23.30% | 26.70% | 20% |
|  | RTX + External Validation | 83.30% | 83.30% | 90% |
|  | RTX – Classify per spectra | 50.70% | 68% | 64% |
|  | RTX + Classify per spectra | 66.60% | 69.30% | 72% |
|  | RTX – Classify majority | 52% | 68% | 60% |
|  | RTX + Classify majority | 68% | 72% | 72% |
|  |  |  |  |  |
|  |  |  |  |  |
|  |  | **SVM** | **GA** | **QC** |
|  |  |  |  |  |
|  | Peaks Used (m/z) | 3423^0.46^ | 10672^0.2^ | 3451 |
|  |  | 5141^0.52^ | 3220^0.09^ | 7532 |
|  |  | 5260^0.60^ | 6956^0.39^ |  |
|  |  | 6406^0.50^ | 5142^0.23^ |  |
|  |  | 6492^0.46^ | 8797^0.34^ |  |
|  |  | 6638^0.65^ |  |  |
|  |  | 7495^0.84^ |  |  |
| **TSA + 5% bovine blood + 10% FBS** |  | 7532^0.83^ |  |  |
|  |  | 8318^0.48^ |  |  |
|  |  | 8570^0.45^ |  |  |
|  |  | 10242^0.44^ |  |  |
|  |  | 10910^0.65^ |  |  |
|  |  | 15050^0.66^ |  |  |
|  |  |  |  |  |
|  | Recognition Capability | 100% | 99.50% | 80% |
|  | Cross Validation | 94.98% | 93.27% | 78.11% |
|  | RTX – External Validation | 69% | 75% | 73% |
|  | RTX + External Validation | 65% | 73% | 63% |
|  | RTX – Classify per spectra | 80.70% | 69.30% | 75.30% |
|  | RTX + Classify per spectra | 55.30% | 55.30% | 70.70% |
|  | RTX – Classify majority | 86.70% | 73.30% | 86.70% |
|  | RTX + Classify majority | 46.70% | 46.70% | 80% |
|  |  |  |  |  |
|  |  |  |  |  |
|  |  | **SVM** | **GA** | **QC** |
|  |  |  |  |  |
|  | Peaks Used (m/z) | 3972^1.64^ | 2163^0.70^ | 2869 |
|  |  | 4211^0.78^ | 3311^0.14^ | 3972 |
|  |  | 6195^0.74^ | 3972^1.53^ | 4211 |
| **TSA + 5% bovine blood + 10 mmol CaCl** |  | 6797^0.72^ | 9376^1.01^ | 4307 |
|  |  | 7397^0.62^ | 15060^1.06^ | 7397 |
|  |  | 7534^1.08^ |  | 7534 |
|  |  | 11945^0.74^ |  | 10676 |
|  |  | 15060^1.08^ |  |  |
|  |  |  |  |  |
|  | Recognition Capability | 100% | 100% | 98.5% |
|  | Cross Validation | 99.23% | 100% | 96.49% |
|  | RTX – External Validation | 67% | 53% | 80% |
|  | RTX + External Validation | 83% | 99% | 72% |
|  | RTX – Classify per spectra | 67.3% | 59.3% | 48.7% |
|  | RTX + Classify per spectra | 90% | 87.3% | 70.7% |
|  | RTX – Classify majority | 73.3% | 60% | 53.3% |
|  | RTX + Classify majority | 93.3% | 93.3% | 80% |
|  |  |  |  |  |
|  |  |  |  |  |
|  |  | **SVM** | **GA** | **QC** |
|  |  |  |  |  |
|  | Peaks Used (m/z) | 3970^1.22^ | 2906^0.39^ | 3309 |
|  |  | 4338^0.64^ | 3970^1.46^ | 3970 |
|  |  | 5651^0.55^ | 4338^0.48^ | 4209 |
|  |  | 6793^0.60^ | 5058^0.38^ | 4711 |
|  |  | 7530^1.06^ | 7529^1.06^ | 5651 |
|  |  | 13854^0.59^ |  | 6622 |
|  |  | 15054^0.95^ |  | 6793 |
| **TSA + 5% bovine blood + 10% FBS + 10 mmol CaCl** |  |  |  | 7529 |
|  |  |  |  | 8610 |
|  |  |  |  | 15054 |
|  |  |  |  |  |
|  | Recognition Capability | 100% | 100% | 92.05% |
|  | Cross Validation | 96.91% | 100% | 90.47% |
|  | RTX – External Validation | 51% | 41% | 54% |
|  | RTX + External Validation | 98% | 91% | 98% |
|  | RTX – Classify per spectra | 41.30% | 48.70% | 24.6% |
|  | RTX + Classify per spectra | 96.70% | 90.00% | 94.7% |
|  | RTX – Classify majority | 33.30% | 46.70% | 26.7% |
|  | RTX + Classify majority | 93.30% | 86.70% | 93.3% |
|  |  |  |  |  |

Supplementary Table 1: Model parameters and accuracy statistics for RTX phenotype classification of all models developed in this study. Superscript numbers signify relative weights of each peak within the respective SVM and GA models. SVM: support vector machine. GA: genetic algorithm
